# tsRNA in head and neck tumors: Opportunities and challenges in the field

**DOI:** 10.1016/j.ncrna.2024.10.003

**Published:** 2024-10-14

**Authors:** Zhuo wu, Yufeng Xu, Changzeng Zhou, Yongbo Zhang, Jingjing Chen

**Affiliations:** a Department of Otorhinolaryngology-Head and Neck Surgery, The Affiliated Women and Children's Hospital of Ningbo University, Ningbo, 315010, China; b Department of Otorhinolaryngology-Head and Neck Surgery, The Affiliated LiHuiLi Hospital of Ningbo University, Ningbo, 315040, China

**Keywords:** tsRNAs, tRF, tiRNA, ncRNAs, Head and neck tumors

## Abstract

Transfer RNA-derived small RNAs (tsRNAs) are a newly recognized class of small non-coding RNAs that are implicated in a variety of cancers, including head and neck tumors. Studies have identified tsRNAs with differential expression profiles in head and neck malignancies, highlighting their potential as biomarkers for diagnosis and prognosis. Functional analyses show that tsRNAs are involved in regulating critical cellular pathways, including those related to cell proliferation, migration, and metabolic processes. Despite these encouraging insights, there are myriad challenges that must be tackled. In summary, tsRNAs present considerable potential as therapeutic targets and biomarkers in the realm of head and neck tumors, meriting further investigation and clinical application to optimize outcomes in the management of these complex diseases. This literature review synthesizes current research on tsRNAs, tsRNAs hold significant promise as biomarkers and therapeutic targets, with the potential to transform diagnostic and treatment strategies for head and neck tumors, ultimately improving patient outcomes.

## 1 Introduction

**Fig. 1:**
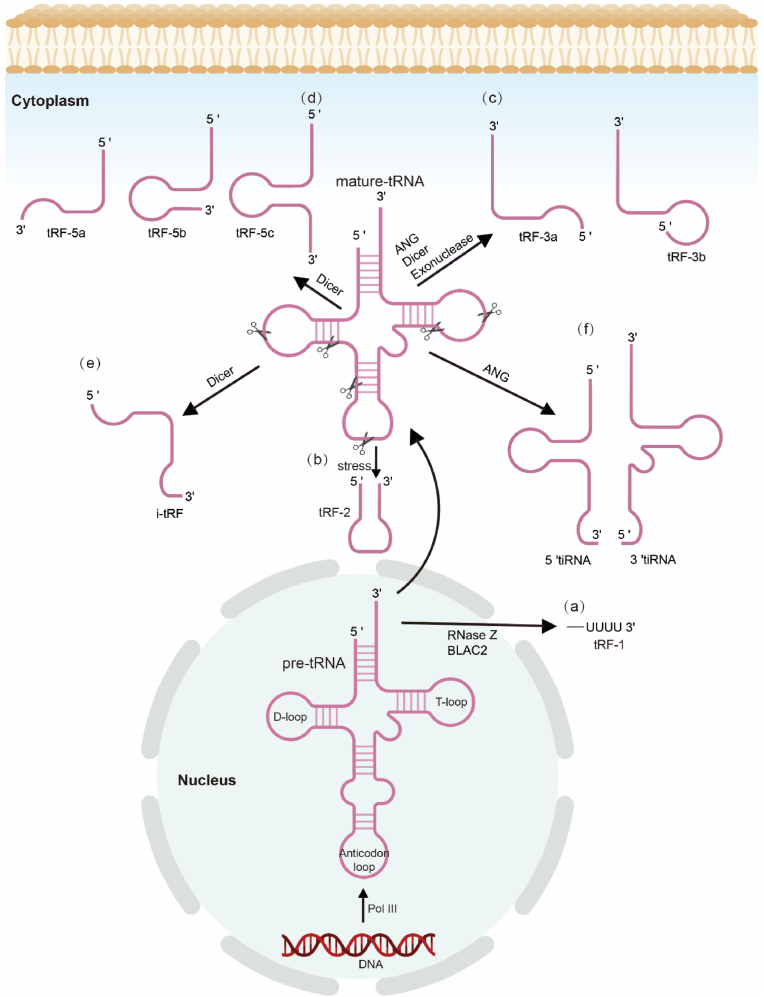
Biogenesis and classification of tsRNAs. Different tsRNA isoforms are generated through enzymatic cleavage by proteins such as Dicer and ANG, leading to distinct subtypes, including tRFs and tiRNAs. (a) tRF-1s are generated through RNase Z- or ELAC2-mediated cleavage of pre-tRNAs in the nucleus. (b) tRF-2 contain the anticodon stem and sequences from both ends of a mature tRNA, but their exact biogenesis remains unclear. (c) tRF-3s originate from the 3′ end of mature tRNAs, are cleaved at the TψC loop by angiogenin (ANG) or Dicer, and can be subdivided into tRF-3a and tRF-3b. (d) tRF-5s are derived from the 5′ end of mature tRNAs and are formed by Dicer-mediated cleavage at the D loop, with further subclassifications into tRF-5a, tRF-5b, and tRF-5c. (e) i-tRFs straddle the anticodon stem and terminate inside both stems, but their biogenesis is still not fully understood. They can be categorized as D-tRF, A-tRF, and V-tRF. (f) tiRNAs, produced by ANG cleavage in the cytoplasm, are classified into 5′-tiRNA, which start from the 5′ end and terminate at the anticodon stem, and 3′-tiRNA, which start at the anticodon stem and extend to the 3′ end of the tRNA.

**Fig. 2:**
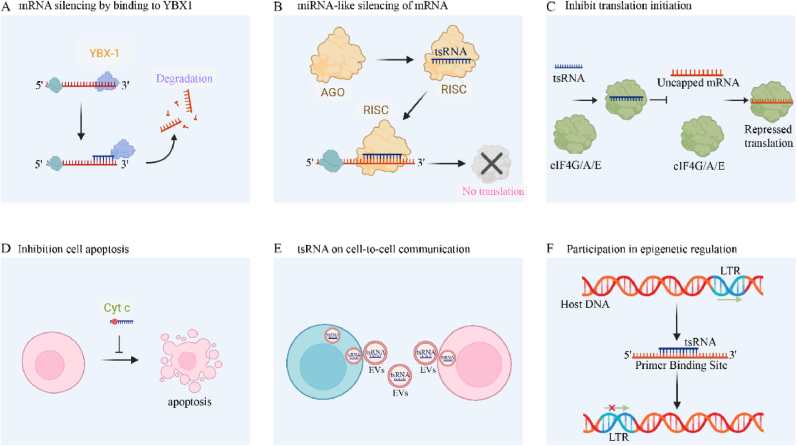
Functions of tsRNAs. (A) tsRNAs regulate gene expression by directly binding to the YBX1 protein, influencing its activity (B) tsRNAs mimic miRNAs by loading into AGO proteins and forming RISC, a complex that regulates gene expression by targeting specific genes. (C) tsRNA interacts with the eIF4 complex, displacing the mRNA binding from eIF4, thereby resulting in the inhibition of translation. (D) tiRNAs directly interact with cytochrome *c, a* key protein in apoptosis. This interaction forms a Cyt C-RNP complex that inhibits the apoptotic process. (E) The packaging of tsRNAs into extracellular vesicles enables these molecules to travel between cells, carrying their gene-regulating potential to distant tissues. (F) tsRNA mitigates the activity of LTR-retrotransposons by specifically targeting the invariant primer binding site that is characteristic of LTR-retrotransposon sequences.

**Fig. 3:**
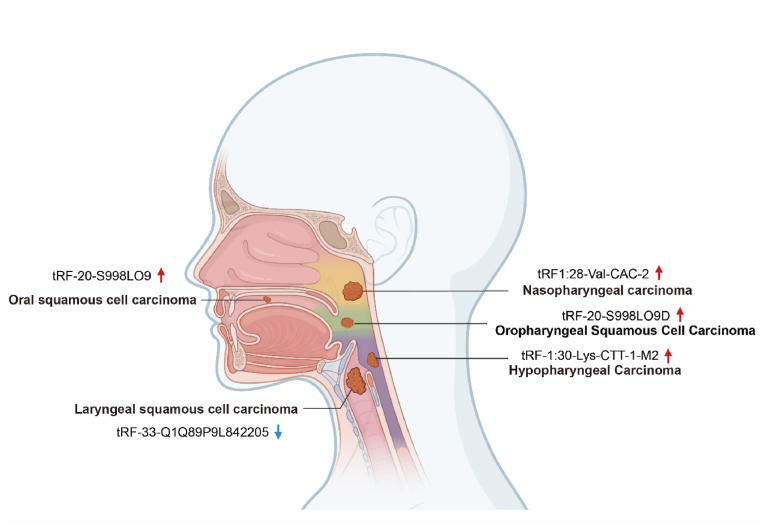
The role of tsRNA in Head and neck cancers. Decreases in the expression of tsRNAs are depicted by blue downward arrows, while increases are indicated by red upward arrows.

Head and neck cancers, encompassing malignancies of the oral cavity, pharynx, larynx, and related sites, represent a significant global health burden [[1], [2], [3], [4]]. Despite advancements in diagnosis and therapeutic interventions, patients diagnosed with head and neck tumors at an advanced stage often face a difficult road ahead in terms of their prognosis [5,6]. The identification and screening of biomarkers are critical for early diagnosis and prognosis assessment in head and neck tumors. As a result, the identification of reliable biomarkers for early diagnosis, prognosis, and therapeutic targeting is critically important to improving patient outcomes (Fig. 1, Fig. 2, Fig. 3).

In recent years, the discovery of non-coding RNAs (ncRNAs) has opened new pathways for understanding the molecular mechanisms driving cancer progression, including head and neck cancers [7,8]. Non-coding RNAs, which do not code for proteins but serve vital regulatory roles, have become increasingly recognized as central players in cancer biology. Among these, tsRNAs have emerged as a novel and significant class of ncRNAs involved in a broad range of cellular processes. Once thought to be mere degradation byproducts, tsRNAs are now understood to be actively produced through specific enzymatic cleavage of precursor or mature tRNAs, in response to various cellular stimuli, including stress and pathological conditions [9].

Non-coding RNAs, including tsRNAs, have emerged as key players in understanding the molecular mechanisms that drive these cancers. Among the various classes of ncRNAs, tsRNAs play key roles in tumor development and progression, making them promising candidates for both diagnostic and therapeutic applications in oncology. The discovery of these biomarkers is a significant breakthrough in head and neck tumor research. These molecular markers are crucial for timely diagnosis, understanding disease progression, and predicting treatment response. The ability to detect head and neck tumors at its earliest stages can dramatically increase the chances of successful intervention and improve overall survival rates.

Initially observed in the urine of individuals with cancer, tsRNAs were originally dismissed as mere degradation byproducts. Now recognize tsRNAs as derivatives of mature or precursor tRNAs, often generated through specific enzymatic cleavage events triggered by various environmental cues. Their widespread distribution, high abundance, stable expression, and tumor-specific expression patterns have positioned them as promising candidates for cancer biomarkers. Based on their cleavage sites and lengths, tsRNAs are classified into two subtypes: tRFs and tiRNAs. tsRNAs exhibit remarkable functional diversity and participate in various regulatory mechanisms. These include acting similarly to microRNAs (miRNAs), modulating viral reverse transcription, influencing DNA transcription, and impacting messenger RNA (mRNA) translation. Emerging evidence highlights the dual regulatory roles of tsRNAs in cancer, demonstrating their ability to either promote or inhibit tumor development under varying conditions. Importantly, a growing body of research has identified substantial differences in tsRNA expression patterns between cancer patients and healthy individuals underscore their potential as diagnostic and prognostic tools [[9], [10], [11]]. Increasing evidence suggests that dysregulation of tsRNAs contributes to cancer initiation, progression, and metastasis [12].

Furthermore, functional studies have commenced elucidating the mechanistic roles of tsRNAs in head and neck tumorigenesis. tsRNAs can regulate gene expression through various mechanisms, such as binding to mRNA targets and modulating their stability or translation, interacting with RNA-binding proteins, and even acting as ceRNAs to sequester microRNAs [[13], [14], [15]]. The regulatory functions of tsRNAs are involved in key cancer-related processes such as cell invasion, apoptosis, and metastasis.

Despite promising findings, the field of tsRNA research in head and neck tumors is still a burgeoning area of research, and many challenges remain to be addressed. The precise mechanisms of tsRNA function in head and neck cancers remain largely unknown. Further research is crucial to elucidate the intricate interplay between tsRNAs and other molecular pathways implicated in tumorigenesis, as well as to validate their clinical utility as therapeutic targets and biomarkers. In this review, we will discuss the biogenesis and classification of tsRNAs, their expression profiles in different head and neck cancers, and their potential roles in tumor initiation and progression. We will also discuss the future perspectives in this rapidly evolving field, with a focus on the translational implications of tsRNA research for improving the diagnosis and prognosis of head and neck cancers.

## 2 Biogenesis and classification of tsRNA

tsRNAs are categorized according to their length and the specific location from which they are derived within the precursor or mature tRNA molecule.

## 3 tRNA-derived fragments (tRFs)

tRFs represent a distinct category of small non-coding RNAs. These molecules, typically 14–30 nucleotides in length, originate from precise enzymatic cleavage events within either precursor or mature transfer RNA (tRNA) transcripts [10,[16], [17], [18]]. The biogenesis of tRFs is a multifaceted process, involving a repertoire of enzymes, including but not limited to Dicer, RNase Z, and angiogenin, each targeting specific regions within the tRNA structure. This results in the generation of distinct tRF subtypes, categorized based on their site of origin and the enzymes responsible for their cleavage [19]. Broadly, tRFs are classified into five main subtypes. tRF-1s are generated from the 3′ end of precursor tRNAs, often carrying a poly-U tail, a hallmark of RNase Z activity. tRF-2s, on the other hand, originate specifically from the loop of mature tRNAs. tRF-3s, a class of tRNA fragments derived from the 3′ end of mature tRNAs, are characterized by the presence of a CCA sequence and are further subdivided into tRF-3a and tRF-3b. Similarly, tRF-5s, cleaved from the 5′ end of tRNAs, exhibit further subclassification (tRF-5a, tRF-5b, and tRF-5c) based on the specific Dicer cleavage site. Finally, i-tRFs, arising from internal regions within mature tRNAs, represent the most diverse subtype with further categorization into six distinct groups. The discovery of tRFs and the unraveling of their biogenesis pathways have turn on new avenues in understanding the complexity of cellular regulation. These molecules have been implicated in various pathological and physiological processes, highlighting their significance in maintaining cellular homeostasis. However, the lack of a standardized nomenclature system for tRFs poses a significant challenge in the field, hindering comparative analyses and potentially masking their full biological relevance. Despite this, the potential of tRFs, particularly in the context of cancer, is undeniable. Further research focused on deciphering their intricate roles in tumor development and progression holds immense promise for advancing diagnostic and therapeutic strategies in the fight against cancer.

## 4 tRNA halves (tiRNA)

tRNA halves, more commonly referred to as tiRNAs, constitute a unique subclass of small RNA fragments with emerging roles in cellular homeostasis and stress response. Unlike other tRNA-derived fragments (tRFs), tiRNAs are predominantly generated under conditions of cellular stress [20]. The biogenesis of tiRNAs is primarily mediated by the endonuclease angiogenin (ANG), which cleaves mature tRNA molecules specifically within the anticodon loop region. This cleavage event generates two distinct tiRNA fragments: a 5′-tiRNA fragment encompassing the 5′ end of the original tRNA and a 3′-tiRNA fragment containing the 3′ end, including the characteristic CCA tail [9,21].

While research on tiRNA functionality is still ongoing, growing evidence supports their involvement in various cellular processes. Similar to microRNAs, tiRNAs can regulate gene expression at the translational level, potentially through interactions with RNA interference machinery [14,22]. Several studies have shown that tiRNAs influence the cellular stress response by modulating the translation of stress-responsive genes or interacting with key pathways involved in cellular adaptation to stress. For example, tiRNAs have been experimentally validated to inhibit global protein translation during stress conditions, aiding cell survival by promoting the formation of stress granules [20,23]. However, while these findings are promising, most experimental validations have been performed in vitro, using cell lines under controlled stress conditions. The limitations of such models, including their inability to fully replicate the complexity of tumor microenvironments, highlight the need for more robust experimental designs. In vivo studies using animal models or patient-derived xenografts are crucial for better understanding the context-specific roles of tiRNAs in cancer progression [24]. Moreover, research methodologies require standardization to ensure reproducibility and comparability across different studies, further underscoring the importance of future investigations into the functional mechanisms of tiRNAs in cancer.

Beyond their roles in translation and stress response, emerging evidence implicates tiRNAs in broader cellular processes, including apoptosis and immune signaling. tiRNAs have been shown to modulate programmed cell death pathways, either promoting or inhibiting apoptosis depending on the specific cellular context and stress stimuli. Additionally, tiRNAs may participate in immune responses, acting as signaling molecules that influence gene expression and modulate immune pathways [[25], [26], [27]]. This highlights the potential for tiRNAs to mediate intercellular communication, particularly in the context of infection or stress.

Intriguingly, tiRNAs have also been found in extracellular vesicles and body fluids, suggesting their potential role as biomarkers for various physiological and pathological conditions. However, to fully understand the mechanisms behind tiRNA packaging, secretion, and uptake, as well as their potential roles in recipient cells, additional research is required [28].

In conclusion, tiRNAs represent a dynamic class of regulatory molecules with emerging roles in translation control, stress response, apoptosis, and immune signaling. Their production under stress conditions and their ability to influence a diverse range of cellular pathways highlight their importance in maintaining cellular homeostasis and responding to environmental changes.

## 5 Functions of transfer RNA-derived small RNAs

### 5.1 Gene regulation

tsRNAs can regulate gene expression through various mechanisms. Some tRFs can bind to Argonaute proteins and function as microRNA-like molecules, targeting mRNAs and inducing translational repression or mRNA degradation [29]. Furthermore, tsRNAs have the capacity to engage with proteins that bind RNA, thereby influencing their functionality and consequently exerting an indirect regulatory effect on gene expression [30,31]. Previous research has shown that that certain CU-box-containing tsRNAs can interact with the oncogenic Y-box binding protein 1. This interaction at the post-transcriptional level results in a modulation of YBX-1 transcript levels [32,33]. Consequently, oncogene mRNA stability is impacted, resulting in altered expression patterns of these cancer-promoting genes.

### 5.2 Protein translation inhibition

tiRNAs emerge as a critical component of the cellular response to a broad spectrum of stressors and can be triggered by environmental and physiological challenges. tiRNAs have been demonstrated to participate in the cellular stress response by regulating protein synthesis, influencing stress granule formation, and impacting cell survival [34]. Some tiRNAs can impede the initiation of translation by displacing the eIF4 complex from the mRNAs, which in turn leads to a decrease in the overall rate of protein synthesis. This mechanism aids in conserving cellular homeostasis during periods of stress [[35], [36], [37]].

### 5.3 Cell apoptosis inhibition

Studies have revealed that tRNAs and their derived small RNAs, particularly tiRNAs, serve as key mediators in the modulation of stress-induced cellular apoptosis. The association of transfer RNAs (tRNAs) with cytochrome C serves to mitigate the cytochrome C-Apaf-1 interaction. This interference consequently hinders the assembly of apoptosomes and suppresses the subsequent activation of caspase-9, key steps in the apoptotic cascade [38,39]. This process ultimately fosters cell survival. Furthermore, angiogenin-induced tiRNAs accumulate during hyperosmotic stress and form RNP complexes with cytochrome C, suggesting a mechanism by which tiRNAs inhibit endogenous apoptosis [40]. Additionally, tiRNAs can selectively augment the translation of mRNAs encoding anti-apoptotic proteins through internal ribosome entry sites (IRES). This mechanism enables cells to withstand adverse conditions and promote survival [41]. These IRES-mediated translations are essential for key regulatory proteins involved in cell survival and apoptosis, especially when cap-dependent translation is downregulated during cellular stress.

### 5.4 Cell-to-cell communication

Extracellular RNA (exRNA) encapsulated within microvesicles and exosomes serves as a crucial signaling molecule in intercellular communication, exerting significant influence on cell behavior [42,43]. These exRNAs, particularly tsRNAs, are abundantly present in extracellular vesicles (EVs) and have emerged as potential diagnostic biomarkers for various diseases due to their stable existence within EVs [44,45]. Studies indicate that immune activation prompts T cells to release EVs enriched with tsRNAs, thereby inhibiting T cell activation and cytokine production [46]. Additionally, the secretion of 5′tiRNA-Gly, derived from tRNA-Gly, increases under acute stress conditions, facilitating intercellular communication. EVs play a pivotal role in mediating cell-to-cell communication, with tsRNAs being notably involved in this process. For instance, activated T cell-derived EVs exhibit high levels of 5′ tRNA-derived fragments (tRFs), and inhibition of these tRFs enhances T cell activation, suggesting a regulatory mechanism where tRFs modulate T cell activation through EV-mediated signaling pathways [46,47].

### 5.5 Participation in epigenetic regulation

While the body strictly regulates transposon activity through various mechanisms, including epigenetic control and the action of tRFs, recent studies have unveiled that tsRNAs can be involved in epigenetic regulation [48,49]. tRFs, specifically tRF-3a and tRF-3b, have been shown to inhibit the activity of LTR-retrotransposons, also known as ERVs in mice are targeted by tsRNAs, which act on their primer binding sites, thereby influencing its expression at the transcriptional and post-transcriptional levels [50]. This highly conserved ability of tRFs to regulate transposon activity suggests their importance as epigenetic regulators, distinct from other effectors that primarily modify DNA or histones to control gene expression and genome stability.

In conclusion, the functions of tsRNAs encompass a wide range of cellular processes, demonstrating their versatility and importance in maintaining cellular homeostasis, tsRNAs have emerged as versatile regulatory molecules with diverse biological functions. As research in this field continues to expand, it is likely that new roles and mechanisms of action for tsRNAs will be discovered, further highlighting their importance in cellular processes and disease pathogenesis.

## 6 The role of tsRNA in head and neck cancers

### 6.1 Nasopharyngeal carcinoma

Nasopharyngeal carcinoma (NPC) remains a serious health challenge, particularly in Southeast Asia [51,52], due to its aggressive nature and the limitations of current diagnostic and treatment modalities [53,54]. The identification of new therapeutic targets and markers is paramount to improving patient care. Recent research has shown the potential role of tsRNAs, in NPC pathogenesis [55]. High-throughput sequencing have revealed distinct tsRNA expression profiles in NPC tissues compared to healthy controls. Specifically, tRF-1:28-Val-CAC-2 and tRF-1:24-Ser-CGA-1-M3 were found to be significantly upregulated, while tRF-55:76-Arg-ACG-1-M2 was downregulated in NPC samples. Bioinformatics analyses suggest that these dysregulated tRFs target genes involved in cancer-associated signaling pathways, further supporting their potential role in NPC development. The altered expression of these tRFs makes them promising candidates for diagnostic and prognostic biomarkers. For example, elevated levels of tRF-1:28-Val-CAC-2 and tRF-1:24-Ser-CGA-1-M3 could potentially serve as indicators for early NPC detection or predict aggressive disease behavior. Conversely, the downregulation of tRF-55:76-Arg-ACG-1-M2 may also hold diagnostic or prognostic value. Beyond their biomarker potential, these dysregulated tRFs represent intriguing therapeutic targets. Strategies to inhibit the oncogenic activities of upregulated tRFs or to restore the function of downregulated tumor suppressor tRFs could offer novel therapeutic avenues for NPC. Functional studies are needed to confirm their specific roles in tumor cell proliferation, apoptosis, metastasis, and immune evasion. Additionally, rigorous clinical validation is necessary to establish their utility as biomarkers and to assess the safety of targeting these tRFs for therapeutic purposes.

In conclusion, tsRNAs, particularly tRFs, are emerging as critical regulators in NPC. Their dysregulation in this malignancy underscores their potential as both diagnostic and prognostic markers and as novel therapeutic targets. Further investigation into their functional roles and clinical translation holds immense promise for improving NPC management and patient outcomes.

### 6.2 Laryngeal squamous cell carcinoma

Laryngeal squamous cell carcinoma (LSCC) continues to pose a significant clinical challenge, often resulting in poor patient outcomes [[56], [57], [58]]. The search for innovative diagnostic and therapeutic strategies has led to a growing interest in the role of tsRNAs in LSCC pathogenesis. Recent research has uncovered a complex interplay between specific tsRNAs and LSCC development, highlighting their potential as both biomarkers [9]. One such tsRNA, tRFTyr, has emerged as a potential oncogene in LSCC. Studies have demonstrated its significant upregulation in LSCC tissues, and mechanistic analyses have revealed its role in promoting tumor growth. tRFTyr appears to enhance the activity of lactate dehydrogenase A (LDHA) by increasing its phosphorylation. This, in turn, leads to elevated lactate production, a hallmark of the metabolic reprogramming often appear in cancer cells, contributing to their enhanced proliferation and survival. Conversely, the 5′-tiRNA fragment tRF-33-Q1Q89P9L842205 exhibits a contrasting role in LSCC [59]. This tsRNA is downregulated in LSCC, and that correlates with advanced disease stage and increased metastatic potential. Functional studies have demonstrated its tumor-suppressive properties, with tRF-33-Q1Q89P9L842205 inhibiting cell metastasis. These effects appear to be mediated through its direct targeting of the phosphoinositide 3-kinase catalytic subunit (PIK3CD), a key signaling molecule implicated in various cancer-related processes. The identification of tsRNAs with opposing roles, such as tRFTyr and tRF-33-Q1Q89P9L842205, underscores the complexity of tsRNA-mediated regulation in LSCC. The distinct expression patterns of these tsRNAs in LSCC tissues suggest their potential utility as markers for early diagnosis, prognostication and predicting treatment response. Furthermore, the functional characterization of these tsRNAs as either oncogenes or tumor suppressors presents exciting opportunities for therapeutic intervention. Strategies to inhibit oncogenic tsRNAs like tRFTyr or to restore the expression or mimic the function of tumor-suppressive tsRNAs like tRF-33-Q1Q89P9L842205 hold significant promise for developing novel LSCC treatments. While promising, the clinical significance of these tsRNAs in LSCC requires further investigation through large-scale studies to confirm their utility as markers and therapeutic targets.

In conclusion, the emerging field of tsRNA research is rapidly expanding our knowledge of LSCC pathogenesis. The identification of tsRNAs with distinct roles in LSCC development and progression, such as tRFTyr and tRF-33-Q1Q89P9L842205, offers promising avenues for developing innovative diagnostic and therapeutic strategies to help the outcomes of patients with this challenging disease.

### 6.3 Oral Squamous Cell Carcinoma

Recent research has focused on profiling these tsRNAs, in both tissue and serum samples from Oral Squamous Cell Carcinoma (OSCC) patients and healthy individuals [60]. One study revealed a significant downregulation of 22 distinct types of 5′-derived tiRNAs in the serum of OSCC compared to healthy controls [61]. Interestingly, while these tRNA halves were detected in both tumor and normal tissues, only the tRNA-Val-CAC-2-1 gene exhibited substantial expression changes in tissue samples and serum. This specific 5′ tRNA half exhibited elevated levels in the serum of OSCC and showed consistent upregulation in tumor tissues. Its consistent dysregulation highlights its potential as a non-invasive biomarker for OSCC diagnosis. The ability to detect this tsRNA in serum is particularly promising, as it offers a readily accessible and minimally invasive approach for OSCC screening and monitoring. However, while these initial findings are encouraging, further research is warranted to validate the clinical utility of that tsRNA as a reliable biomarker for OSCC. Large-scale clinical studies are necessary in detecting OSCC at different stages and to evaluate its potential for predicting disease progression and treatment response. Moreover, mechanistic studies are crucial to elucidate its role in OSCC pathogenesis to understanding its molecular targets and downstream effects.

In conclusion, the identification of dysregulated tsRNAs (5′ tRNA-Val-CAC-2-1 half) in OSCC is very helpful to improve early diagnosis and developing innovative treatment strategies. However, further research is essential to translate these findings into clinical applications that can ultimately improve patient outcomes.

### 6.4 Oropharyngeal squamous cell carcinoma

Oropharyngeal squamous cell carcinoma (OPSCC) is a diverse disease with variable prognosis [62,63]. While traditional clinical parameters offer some guidance, more precise and reliable biomarkers are needed to improve prognostication and guide treatment strategies. Recent research has highlighted the potential of tRFs as key players in OPSCC development and progression. Identified a specific tRF, tRF-20-S998LO9D (tRF-20), as a particularly promising prognostic marker in OPSCC [64]. Elevated tRF-20 expression in tumor tissues is significantly associated with poorer overall survival, independent of age, HPV status, and disease stage. This suggests that tRF-20 could provide valuable information for risk stratification and personalized treatment decisions. While the precise mechanisms by which tRF-20 contributes to OPSCC progression remain to be fully elucidated, bioinformatics analyses suggest a potential interaction with the translation initiation factor eIF4B. This interaction could disrupt normal protein synthesis and lead to the uncontrolled cell growth and proliferation that characterize cancer. The discovery of tRF-20 as a potential prognostic biomarker and its potential role in OPSCC pathogenesis turn on exciting new avenues for improving patient outcomes. Additional studies are necessary to validate these findings in larger patient cohorts and to develop effective strategies to target tRF-20 for therapeutic benefit. This could involve developing antisense oligonucleotides or small molecule inhibitors to suppress tRF-20 expression and inhibit tumor growth.

### 6.5 Hypopharyngeal carcinoma

Hypopharyngeal carcinoma (HPC) presents a significant clinical challenge due to its aggressive nature and poor prognosis [65]. Utilizing high-throughput sequencing of tRNAs extracted from extracellular vesicles (EVs) of healthy individuals and HPC patients, identified a panel of tRFs. Among these, tRF-1:30-Lys-CTT-1-M2 exhibited significant upregulation in HPC and demonstrated a strong association with clinicopathological features [66]. Elevated expression of the tRF was significantly correlated with advanced tumor stage, higher grade of differentiation, and patient history of smoking and alcohol consumption, all recognized risk factors for aggressive HPC. Notably, Cox regression analysis revealed that tRF overexpression, along with advanced tumor stage (III-IV), smoking, and alcohol consumption, were irrelevant factors in HPC. These results show that tRF plays a key role in HPC progression and metastasis. This tRF may contribute to the molecular mechanisms driving tumor development and could potentially serve as a novel, non-invasive biomarker for HPC diagnosis and risk stratification for lung metastasis. Further investigation into the functional role of tRF in HPC pathogenesis is warranted. Understanding its mechanism of action could help researchers design more effective treatments for this aggressive disease by identifying promising therapeutic targets.

## 7 Conclusion and future perspectives

This review has provided a comprehensive overview of the role of tsRNAs in head and neck tumors, highlighting their potential as biomarkers and therapeutic targets. The functional diversity and involvement of tsRNAs in key cellular processes, including cell proliferation, apoptosis, invasion, and metastasis, underline their importance in tumor biology. Despite these promising findings, several challenges must be addressed to fully exploit the diagnostic and therapeutic potential of tsRNAs in head and neck cancer management.

One of the most significant opportunities presented by tsRNAs is their potential to serve as biomarkers for early diagnosis and prognosis. Several studies have demonstrated differential expression patterns of tsRNAs in various cancers, including head and neck malignancies. However, translating these findings into clinical practice presents several challenges. For example, although tsRNAs show promise as biomarkers, the specificity of certain tsRNA species for particular cancer types remains unclear. More research is required to determine whether the changes in tsRNA expression are exclusive to head and neck tumors or if they occur in other malignancies as well. Additionally, standardizing detection methods for tsRNAs, such as optimizing assays for sensitivity and reproducibility, is a crucial step toward ensuring their widespread clinical application. Furthermore, while several tsRNAs have been shown to correlate with disease severity and patient outcomes, large-scale cohort studies are necessary to validate their prognostic value. Without larger sample sizes and standardized experimental protocols, the diagnostic potential of tsRNAs remains limited. Therefore, future studies should focus on incorporating more diverse patient populations and uniform methodologies to ensure that the findings are broadly applicable and reproducible across different clinical settings.

Although significant progress has been made in understanding the biogenesis and function of tsRNAs, several mechanistic aspects remain to be elucidated. For instance, the precise molecular mechanisms by which tsRNAs regulate gene expression, particularly through interactions with RNA-binding proteins or microRNAs, are not fully understood. Additionally, more robust experimental designs are required to validate the regulatory roles of tsRNAs in cancer development, as current studies are often limited by in vitro models that may not fully capture the complexity of tumor microenvironments.

The potential of tsRNAs to act as ceRNAs and their involvement in the regulation of key cancer-related pathways are particularly intriguing areas for future research. Decoding these complex interactions could provide critical insights into how tsRNAs influence tumor progression and response to treatment. Furthermore, investigating the role of tsRNAs in epigenetic regulation, such as their interactions with transposons and other genomic elements, represents an emerging frontier in the field. These mechanisms could uncover novel targets for therapeutic intervention.

The therapeutic implications of tsRNAs, though still in the early stages of exploration, offer a promising avenue for cancer treatment. Several tsRNAs have demonstrated either oncogenic or tumor-suppressive properties, making them potential targets for drug development. For instance, inhibiting oncogenic tsRNAs or restoring the function of tumor-suppressive tsRNAs could lead to novel therapeutic strategies for head and neck tumors. However, the delivery, specificity, and safety of such tsRNA-based therapies remain major challenges that must be addressed before clinical translation. One of the key obstacles in developing tsRNA-targeted therapies is the delivery of therapeutic molecules to tumor sites while minimizing off-target effects. The use of nanoparticle-based delivery systems or other advanced platforms may offer potential solutions, but these approaches need to be rigorously tested in preclinical and clinical settings to ensure their efficacy and safety. Furthermore, regulatory hurdles, including FDA approval and the long-term monitoring of potential side effects, should also be considered when developing tsRNA-based therapies.

In conclusion, tsRNAs represent a promising class of biomarkers and therapeutic targets in the field of head and neck cancer. Their unique ability to regulate key cellular processes and their differential expression patterns in tumors position them as valuable tools for improving diagnostic and treatment strategies. However, overcoming the challenges related to their clinical application and understanding their precise mechanisms of action will be critical for fully harnessing their potential. Future research should focus on validating the clinical utility of tsRNAs in larger, more diverse cohorts and developing effective therapeutic interventions that target specific tsRNA pathways. With further investigation, tsRNAs could play a transformative role in the management of head and neck tumors, ultimately improving patient outcomes.

## CRediT authorship contribution statement

**Yufeng Xu:** Writing – original draft. **Changzeng Zhou:** Validation, Data curation. **Yongbo Zhang:** Supervision, Data curation. **Jingjing Chen:** Project administration, Conceptualization.

## Declaration of competing interest

The authors declare that they have no known competing financial interests or personal relationships that could have appeared to influence the work reported in this paper.
